# Co‐Producing Equitable Perinatal Mental Health Care: Facilitators and Barriers to Access Among Underserved Women in the PRAMS Study

**DOI:** 10.1111/hex.70766

**Published:** 2026-07-12

**Authors:** Elena Sheldon, Naseeb Ezaydi, Danielle Hahn, Kelly Hobbs, Saima Ahmed, Helen Miles, Julia Thompson, Katie Marvin‐Dowle, Kate Fryer, Laura Sutton, Victoria Silverwood, Caroline Mitchell, Daniel Hind, Kelly Mackenzie

**Affiliations:** ^1^ Sheffield Health Partnership University NHS Foundation Trust Sheffield UK; ^2^ School of Medicine and Population Health The University of Sheffield Sheffield UK; ^3^ LIGHT Peer Support Sheffield UK; ^4^ Public Health, Sheffield City Council Sheffield UK; ^5^ Public Health, Doncaster Council Doncaster UK; ^6^ School of Medicine Keele University Keele UK; ^7^ Faculty of Medicine and Health University of Leeds Leeds UK

**Keywords:** complex intervention development, co‐production, mental health, perinatal, underserved groups

## Abstract

**Introduction:**

Underserved women experience disproportionately high rates of perinatal mental health (PMH) difficulties but face substantial barriers to accessing appropriate care. Evidence on how services can be redesigned to improve equitable access remains limited. The Perinatal Redesign for Accessing Mental Health Services (PRAMS) study aimed to co‐produce an intervention to improve access to PMH support for underserved women and birthing people.

**Methods:**

PRAMS is a mixed‐methods study informed by Accelerated Experience‐Based Co‐Design and the Medical Research Council framework for complex intervention development. This paper reports findings from Work Packages 1 and 2. Work Package 1 included a national survey of professionals (*n* = 129) and semi‐structured interviews (*n* = 19) exploring service provision and barriers to care. Work Package 2 involved 10 focus groups and 4 interviews with underserved women and birthing people (*n* = 50) recruited through Community Research Link Workers with lived experience. Data were analysed using framework analysis guided by the Candidacy Framework.

**Results:**

Barriers to PMH care were identified across all domains of candidacy. Structural barriers included fragmented services, unclear referral pathways, and limited resources. Cultural stigma, language barriers and fears regarding child protection services limited help‐seeking. Trust, continuity of care and community‐based services facilitated engagement. Both professionals and women described a mismatch between rigid service models and women's preferences for relational, flexible and culturally responsive support.

**Conclusion:**

Access to PMH care among underserved populations is shaped by complex structural and relational factors. Findings highlight the need for flexible, community‐based and relationship‐centred models of care. Co‐production with underserved communities offers a promising approach for developing equitable PMH services to better meet the needs of women facing multiple vulnerabilities.

## 1 Introduction

Perinatal mental health (PMH) problems are the most common complication of childbearing, affecting approximately 10%–20% of women during pregnancy and the first year post‐partum [1]. These difficulties can have profound and lasting impacts on women, infants, and families, including adverse effects on maternal wellbeing, parent‐infant relationships and early child development [2, 3]. In the United Kingdom, the economic and societal costs of PMH problems are estimated at £8.1 billion annually, with approximately £1.2 billion borne by the National Health Service (NHS) [4]. Suicide remains the leading cause of maternal death in the year following childbirth, highlighting the serious consequences for UK women of unmet mental health needs during the perinatal period [5]. Internationally, PMH problems are increasingly recognised as a major public health concern and contributor to maternal morbidity, particularly among socially and economically marginalised populations [6]. Internationally, PMH problems are increasingly recognised as a major public health concern and contributor to maternal morbidity, particularly among socially and economically marginalised populations [6].

Despite this substantial burden, global evidence highlights persistent inequalities in access to culturally responsive PMH care across both high‐ and low‐resource settings [7, 8]. Women facing multiple forms of disadvantage, such as poverty, ethnic marginalisation, limited English proficiency, and trauma histories, experience disproportionately high rates of perinatal mental illness but are among the least likely to access effective support [9, 10]. Evidence from the Mothers and Babies: Reducing Risk through Audits and Confidential Enquiries across the UK (MBRRACE‐UK) 2025 report [11] shows that mortality and severe mental illness are more common among women living in deprived areas and among those from minoritised ethnic groups, with Black women being three times more likely to die than White women in the year after childbirth. Similar disparities have been reported internationally among migrant women, racialised communities, women affected by displacement, and those experiencing poverty or social exclusion, suggesting that inequalities access to PMH care among underserved groups is a widespread global challenge [10]. Women with multiple vulnerabilities, including substance misuse, learning disabilities, neurodivergence, or physical health comorbidities, may also fall between service thresholds, where mental health needs are considered too complex for primary care psychological therapies, but not severe enough for secondary mental health services [12].

The perinatal period represents a critical window of opportunity for early intervention and therapeutic change. Despite routine contact with healthcare services during the perinatal period, referral rates to PMH care remain low, and even fewer engage with treatment [13, 14]. Previous research consistently demonstrates that access to PMH care is shaped by interconnected structural, cultural and relational barriers. [15, 16, 17, 18]. Across diverse settings, women report difficulties recognising symptoms, fears of stigma and child protection involvement, limited continuity of care, fragmented referral pathways, and services that are insufficiently culturally responsive or trauma‐informed [15, 16, 17, 18, 19, 20]. Studies also consistently highlight the importance of trusted relationships, flexible models of care, peer support and community‐based approaches in facilitating engagement with services [15, 17, 18, 19, 20]. Together, these factors can delay recognition of need and prevent women from accessing timely and appropriate support. Despite this growing evidence base, much of the literature has focused on describing barriers to access rather than understanding how services can be redesigned collaboratively with underserved communities to address persistent inequalities in care.

Although international and national policy initiatives have prioritised improvements in PMH services and aimed to expand provision across the care pathway [6, 21, 22], practical guidance on how services can be redesigned to address persistent inequalities remains limited. Recent studies have demonstrated the potential effectiveness of culturally adapted psychological interventions for specific populations, such as South Asian women with postnatal depression [23]. However, such interventions are often condition‐specific, population‐limited, and difficult to integrate into routine care systems.

Addressing inequalities in PMH therefore requires approaches that move beyond single interventions to consider how systems are structured, delivered and accessed. Consistent with the Medical Research Council (MRC) framework, this includes the development of interventions that are explicitly designed to operate within, and influence, complex care systems, rather than functioning as isolated components of care [24]. In this context, early consideration of implementation is critical to ensure that newly developed approaches are feasible, acceptable, and sustainable within real‐world care settings. Implementation science frameworks can support this process by identifying multilevel determinants of service delivery and uptake, including organisational, professional, and contextual factors.

One important limitation of existing research is that underserved populations have historically been underrepresented in both health research and service design. Structural power imbalances [21], distrust in institutions, and legacies of cultural trauma [25, 26] can limit the participation in research and reduce opportunities for communities to influence the services intended to support them. As a result, interventions and service models may fail to reflect the lived experience realities and priorities of the populations most affected by inequalities in care.

Co‐production offers a promising approach to addressing these challenges by actively involving people with lived experience in the design, development and evaluation of healthcare services [27]. Through collaborative processes that value experiential knowledge alongside professional expertise, co‐production can support the development of more accessible, culturally responsive and contextually grounded models of care. Co‐production approaches have also demonstrated potential to improve the relevance, acceptability and implementation of interventions among underserved populations across a range of healthcare settings [28, 29, 30]. There remains limited evidence however, on how implementation‐informed and co‐produced approaches can be used to redesign PMH services for underserved women within routine healthcare systems.

The Perinatal Redesign for Accessing Mental Health Services (PRAMS) study [31] is a mixed‐methods co‐production study designed to address these gaps by combining implementation science and lived experience perspectives to inform the development of an intervention aimed at improving access to PMH care for underserved women and birthing people in Sheffield and Doncaster. It contributes methodologically through in‐depth qualitative work and innovative partners with Community Research Link Workers and VCSE organisations, bringing together diverse perspectives and translating findings into an intervention for more equitable and culturally responsive PMH services. Unlike many previous studies focused on signle populations or specific interventions, PRAMS adopts a systems‐informed and co‐produced approach across multiple underserved groups and care settings.

This paper reports findings from the first two stages of the study, which aimed to explore professional perspectives on PMH service provision (Work Package 1) and to understand the lived experiences of underserved women and birthing people with PMH needs (Work Package 2). Findings from Work Packages 1 and 2 will provide the basis for co‐designing an intervention that is feasible, acceptable, and capable of being integrated within existing perinatal care systems, informed by implementation science principles. The intervention co‐design phase (Work Package 3) will be reported separately.

## 2 Aims and Objectives

The overall aim of the Perinatal Redesign for Accessing Mental Health Services (PRAMS) study was to co‐design an experience‐based intervention for underserved women and birthing people with unmet perinatal mental health needs. The objectives were to: (1) explore professional experiences of delivering perinatal pathways and identify perceived barriers and facilitators for underserved groups; (2) understand the lived experiences of underserved women and birthing people with perinatal mental health needs; and (3) co‐design a systems‐informed, experience‐based intervention with these groups.

## 3 Materials and Methods

### 3.1 Design

**Figure 1:**
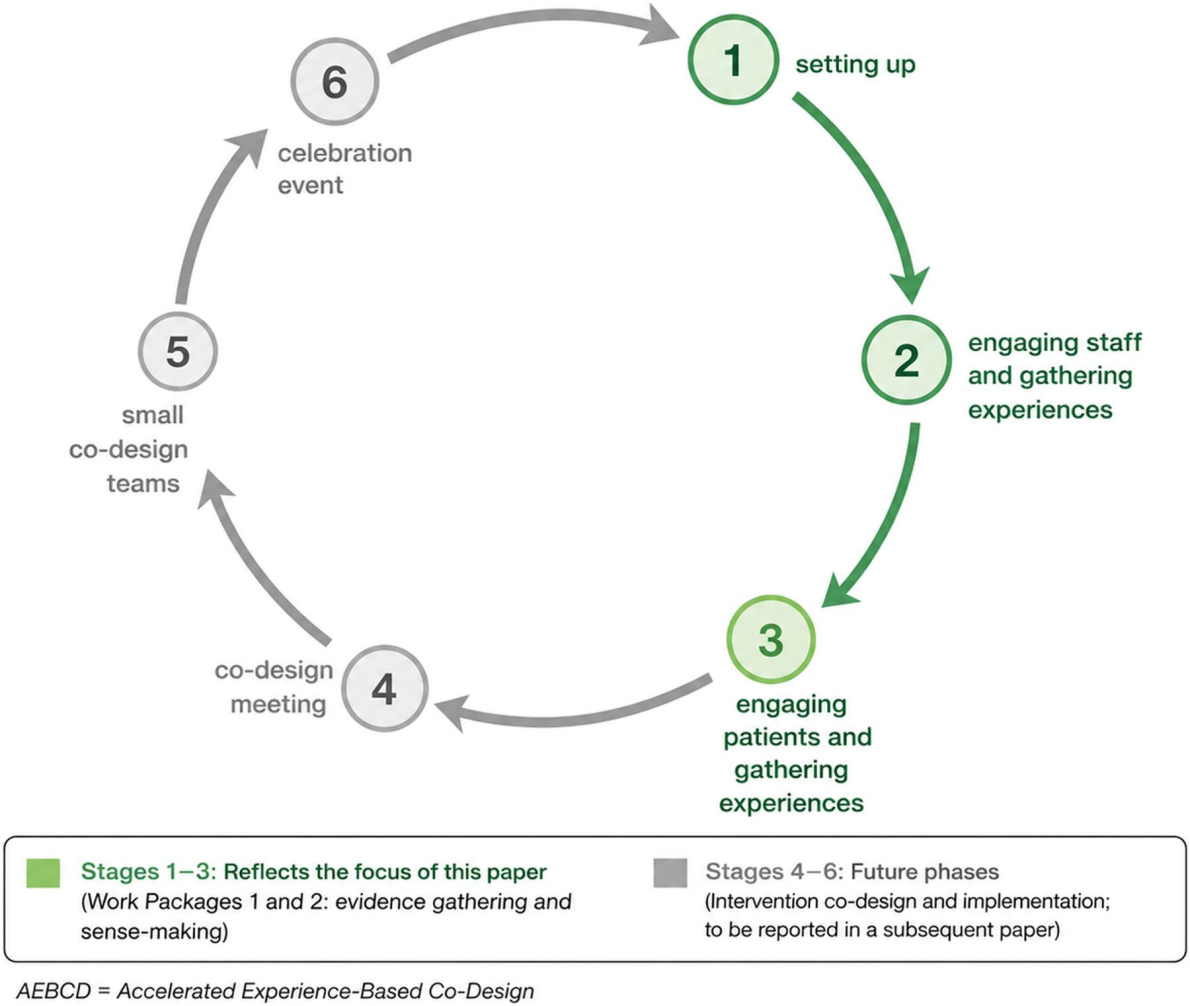
Stages 1–3 of the AEBCD Framework.

PRAMS was a mixed‐methods study comprising three work packages informed by a modified version of the six‐stage Accelerated Experience‐Based Co‐Design (AEBCD) framework [32] (Figure 1) and MRC guidelines for complex intervention development [24].

AEBCD integrates participatory action and service improvement to redesign patient and provider experiences of care [32]. The AEBCD framework informed the overall co‐design process, providing a structured approach to engaging stakeholders and integrating experiential knowledge into intervention development. Its emphasis on meaningful involvement of both professionals and underserved women ensured that the resulting intervention reflects priorities grounded in lived experience and service delivery contexts.

Modifications to the original framework were made to accommodate constraints associated with working within existing systems and research governance structures, and to address stigma and mistrust of research among underserved communities. Adaptations to the framework included flexible approaches to recruitment and participation, use of online and community‐based venues to gather staff and lived experiences, removal of ‘trigger film’ production, and iterative co‐design workshop formats to support psychological safety, accessibility, and sustained engagement.

The study design aligned with core elements of the MRC framework, including consideration of context, stakeholder engagement, refinement of programme theory, and identification of uncertainties. The MRC framework provided methodological structure to the study design and ensured that the emerging intervention was theoretically grounded, evidence‐informed, and appropriate for implementation within complex healthcare systems.

This paper reports on stages 1–3 of the AEBCD process, corresponding to Work Packages 1 and 2, conducted between January and September 2025. The study follows SQUIRE 2.0 reporting standards and EBCD reporting recommendations (see Supporting File 1).

### 3.2 Setting and Participants

#### 3.2.1 Work Package 1

An online survey was developed for professionals working in a paid or voluntary capacity with women and birthing people during the perinatal period, including those in mental health, maternity, primary care, and community services. Initial recruitment focused on Sheffield and Doncaster, with subsequent expansion to a national sample following an ethics‐approved protocol amendment. Sample characteristics were based on professional roles and organisation to ensure breadth of professional experiences in delivering care to a wide range of underserved women and birthing people across the perinatal journey.

A non‐probability snowball sampling approach was used. Recruitment was primarily conducted through professional networks andexisting collaborations within the Start for Life Programme. The survey was promoted via presentations to relevant professional steering groups, where a QR code and hyperlink were shared and attendees encouraged to disseminate these within their own networks. Members of the project management group further circulated the survey through professional communication channels (such as online forums and messaging groups). In addition, two NHS research partner organisations were established as recruitment sites and distributed email invitations, including the participant information sheet and survey link, to relevant clinical departments. Participants were invited to opt in to a follow‐up interview through the consent form. A purposive sample was selected to ensure diversity in professional roles, service settings, and experience of PMH care. Semi‐structured interviews explored perceived barriers to PMH care for underserved populations and existing service provision. The topic guide was informed by the Consolidated Framework for Implementation Research (CFIR) [33] and the Candidacy Framework [34].

**Figure 2:**
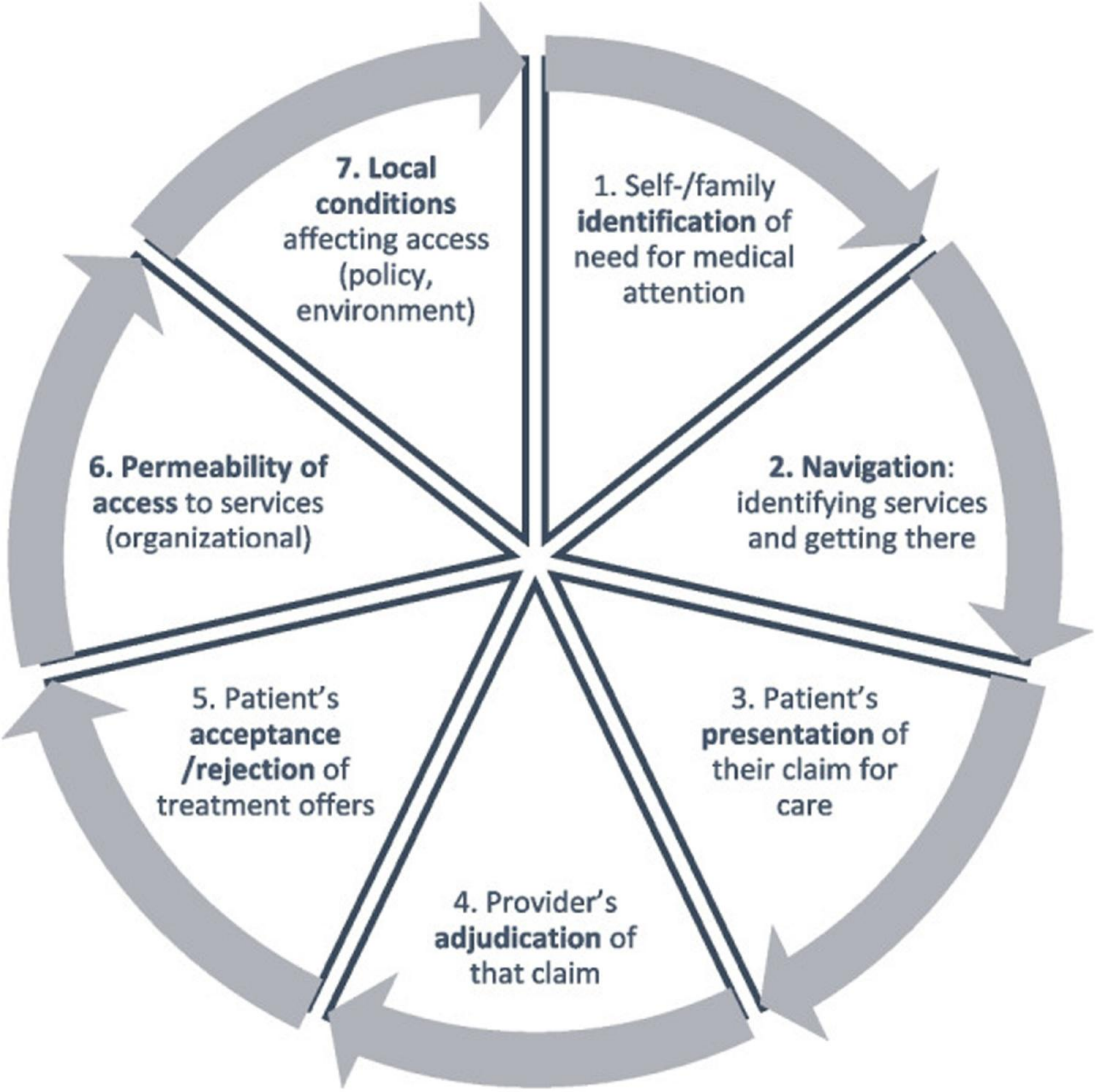
The seven dimensions of the candidacy framework.

The Candidacy Framework conceptualises access to healthcare as a dynamic process negotiated between individuals and services, particularly among underserved populations [34, 35]. Rather than a single point of entry, access is shaped through interactions across several domains, including identification of need, navigation of services, permeability of services, professional adjudication, and engagement with care (Figure 2). These domains capture how eligibility for care (“candidacy”) is recognised, negotiated and enacted over time.

#### 3.2.2 Work Package 2

This work package used the *IBISES* Community Research Link Worker (CRLW) model [36, 37] to recruit and train four women with lived experience of perinatal mental health who were already embedded within existing local underserved community groups. Each CRLW represented a different ethnic background and was embedded within local underserved community groups: South Asian, Arab, Black African, and Somalian. CRLWs received training in research ethics, focus group facilitation, qualitative analysis, PMH presentations, and safeguarding. They were remunerated for their contributions to participant recruitment and analysis.

CRLWs used purposive sampling to recruit women and birthing people through LIGHT Peer Support and Start for Life community groups delivered across the Family Hub centres in Sheffield and Doncaster. Recruitment also took place at Emotional Wellbeing Clinics, led by Perinatal Mental Health Midwives and the Family Intervention Service (FIS), targeting antenatal women between 15 and 25 weeks' gestation in areas of high deprivation and ethnic diversity. Recruitment settings were specifically selected due to their established role in supporting underserved women and families. CRLWs also recruited through trusted pre‐existing community relationships and networks developed through their professional and lived experience roles.

To reduce digital exclusion, recruitment was conducted exclusively in person. This approach was intentionally prioritised to facilitate inclusion of women who may experience barriers to digital engagement, including limited internet access, language barriers, low digital confidence, or lack of formal contact details. Flexible options for subsequent study participation were then offered, including online and remote engagement where appropriate.

Eligible participants were aged 16 or older, with self‐reported current or past PMH difficulties, and identified with one or more protected characteristics defined by the Equality Act, 2010.

CRLWs introduced the study during groups or following clinic appointments. With verbal consent, an Expression of Interest (EOI) form was completed and securely shared with the research team. Potential participants were subsequently contacted by a researcher (DH or NE) to confirm eligibility, provide a Participant Information Sheet, and arrange a focus group or interview.

### 3.3 Data Collection

#### 3.3.1 Work Package 1

A cross‐sectional online survey was developed and hosted in Qualtrics (Provo, UT, USA), incorporating the Pragmatic Context Assessment Tool (pCAT) [38], which is grounded in the CFIR and designed to assess implementation barriers. The pCAT was selected for its focus on capturing contextual barriers and facilitators to implementation in real‐world healthcare settings, making it well‐suited to assessing the introduction of a new PMH intervention. The pCAT has demonstrated good face validity and usability in its development through testing with healthcare professionals, supporting its relevance for applied implementation research. The survey also collected demographic and professional background data. Usability testing was conducted with two non‐specialist team members (D.H., E.S.).

The survey was open from 20/02/2025 and re‐opened from 09/07/2025 to 08/08/2025 for targeted recruitment. Eligibility was confirmed via a screening form embedded in the survey. Ineligible responses (where fully informed consent had not been given) were excluded from analysis, after quantifying the full response rate. Responses submitted by staff members who were testing the survey in the live environment, rather than the designated test version, were also removed from the dataset. The participant information sheet was provided and consent to participate confirmed prior to completion of the survey.

#### 3.3.2 Work Package 2

Ten focus groups were organised according to participants’ underserved identity and shared experiences, including first language, mental health history, or community affiliation. Focus groups typically lasted 2 h, including refreshment breaks. Where group attendance was not feasible, four participants were offered individual 1‐h interviews by phone, online, or in person. Sessions were held in accessible, child‐friendly venues including local mosques, Family Hubs and home visits, during school term time to reduce childcare barriers. CRLWs assisted with engagement, cultural context, and language needs. Venues were identified by CRLWs in line with participant preferences (e.g., women‐only spaces within local community settings such as mosques), and interpreters were arranged where required to support participation of non‐English speaking women, including a Farsi interpreter and a Somalian interpreter. Informed consent was obtained at the start of each session by a trained researcher (D.H. or N.E.), supported by a clinical facilitator (K.H. or E.S.) and the community engagement lead (S.A.). Focus groups and interviews were guided by a semi‐structured topic guide based on the Candidacy Framework, covering perceived causes of perinatal mental health issues, access barriers, prior support received, and preferred types of care. Interpreters or bilingual CRLWs were present where required.

Audio recordings were used to collect qualitative data using encrypted devices, uploaded to a secure drive, and deleted from the recorder. Transcripts were typed by external transcribers and fully anonymised prior to analysis. Field notes were typed and stored securely as part of data collection; handwritten notes were transcribed and destroyed.

### 3.4 Analysis

#### 3.4.1 Work Package 1

Survey data were analysed using descriptive statistics and interpreted according to CFIR guidance. There were no continuous variables collected. Categorical data were presented as frequency counts with percentages calculated using a denominator of those with non‐missing response. Missing data were quantified. Recurring responses entered as free text under ‘other’ were categorised and added to summary tables; remaining free text responses were summarised in text. The pCAT data were coded as per instrument guidance.

Free‐text responses were categorised where appropriate, and all free‐text entries were reviewed to identify and redact any potentially identifiable information; however, no such information was detected. Aside from these cleaning procedures, the data remained unaltered. For transparency, the number of respondents to each survey item was recorded and reported.

The quantitative analysis was primarily descriptive and exploratory, designed to identify perceived barriers and facilitators to introducing a new perinatal mental health intervention. Survey findings were used analytically to inform understanding of the service context, identify priority implementation domains (such as leadership engagement, resource constraints), and support triangulation with qualitative data. The survey was therefore not intended to generate standalone inferential findings, but to complement and contextualise the in‐depth qualitative analysis within the mixed‐methods design.

Interview transcripts were independently coded by two researchers using Framework Analysis [39] within NVivo (QSR International), following the stages of familiarisation, theme identification, indexing, charting, and mapping. This process involved initially reading the transcripts so that the researchers could familiarise themselves with the raw qualitative interview data. The researchers then used codes based on the CFIR domains and the stages of the Candidacy Framework as a system for marking sections of the transcripts that were of special interest. Codes were then converted into themes according to the core concepts that they represented and to reflect the most important aspects of the findings. Coding was conducted manually and organised in Excel spreadsheets to facilitate comparison across groups (such as professional groups) and mapping to the different frameworks. Themes were iteratively refined through team discussions to produce the final framework. Secondary reviewers double coded 47% of the transcripts.

The Consolidated Framework for Implementation Research (CFIR), operationalised through the pCAT tool, informed analysis of professional data by providing an a priori structure to identify organisational and contextual determinants of implementation. This supported systematic interpretation of barriers and facilitators within service delivery settings.

#### 3.4.2 Work Package 2

Focus group and interview transcripts were analysed using Framework Analysis by three independent coders, and two CRLWs. Qualitative analysis training was provided to the CRLWs prior to analysis activities. Secondary reviewers double coded 43% of the transcripts. Second reviewers included professionals with both clinical and academic research backgrounds, including an NIHR Academic Clinical Lecturer, an Assistant Psychologist, and a Professor of General Practice Research. Thematic development was guided by the Candidacy Framework but allowed for inductive theme generation. The same process for analysis was followed as described for Work Package 1 interviews. The Candidacy Framework guided the analysis of qualitative data from women and birthing people by conceptualising access to care as a dynamic process negotiated between individuals and services. It enabled interpretation of lived experiences in relation to how eligibility, navigation, and engagement with PMH services are constructed over time.

Following Francis et al. [40], we specified a priori that a combination of at least six focus groups and interviews would be analysed before considering saturation, allowing for stopping after every two further interviews or focus groups if two researchers agreed that no new themes were identified. Qualitative data collection ran ahead of analysis which, retrospectively, showed that data saturation was achieved in the first four focus groups and two interviews. The remaining focus groups and interviews were included in the analysis to further explore themes across the perinatal journey and between different underserved groups. The sample size (n = 50) was in line with methodological literature that recommends 9–17 interviews as generally sufficient for data saturation [41].

### 3.5 Safeguarding

Given the sensitivity of perinatal mental health discussions, safeguarding procedures were implemented to protect both participants and staff. An ethically approved risk policy and safeguarding plan was followed in line with NHS and University of Sheffield procedures (see Supporting Files S2 and S3).

No formal structured clinical risk assessment or suicidality screening tool was used as part of eligibility screening.

Participants were eligible to take part if they met the inclusion criteria described above; however, if individuals presented during recruitment activities with obvious acute clinical need or distress that suggested immediate risk, participation was deferred and signposting was prioritised.

CRLWs received training from a Clinical Psychologist and Assistant Psychologist on recognising perinatal mental health risk factors (“red flags”), safeguarding concerns, and implementation of the study risk protocol during community engagement and research activities. Psychologically informed practices, such as wellbeing check‐ins and check‐outs for the CRLWs and research team, were embedded throughout study activities.

Focus groups and interviews were co‐facilitated by clinical staff working in local services and, where unmet mental health needs were identified during recruitment or data collection, researchers followed the risk and safeguarding protocol. This included supportive discussion, signposting, and escalation through appropriate clinical or safeguarding pathways where required, including communication with participants’ GPs where clinically indicated and consistent with the consent process. Where needs fell between primary and secondary care, the clinical team consulted with local mental health and third sector services to ensure appropriate referral pathways were available. Participation in the study did not automatically lead to exclusion or removal of data following disclosure.

### 3.6 Intervention Development

Findings from Work Packages 1 and 2 aligns with the experiential evidence‐gathering and sense‐making phases of the AEBCD framework, which emphasise understanding staff and patient experiences as the foundation for collaborative service redesign. The subsequent co‐design and implementation phase (Stages 4–6), in which stakeholders collaboratively develop a programme theory and refine intervention components, will be reported in a separate paper.

## 4 Results

### 4.1 Work Package 1

The online survey was accessed 286 times. Of those, 129 (45.1%) gave full consent and of those 113 (87.6%) contributed at least one response to the pCAT, all of whom confirmed they had a role supporting birthing people in the perinatal period (Table 1). The job roles with the hgighest frequency of response were Health Visitor (16.8%), Midwife (13.9%), General Practitioner (11.9%) and Family Intervention Worker (9.9%).

**Table 1:** Job roles of professionals surveyed.

Job title	Frequency
Health Visitor	17 (16.8%)
Midwife	14 (13.9%)
General Practitioner	12 (11.9%)
Family Intervention Worker	10 (9.9%)
Clinical Psychologist	7 (6.9%)
Mental Health Nurse	5 (5.0%)
Cognitive Behavioural Therapist	4 (4.0%)
Parenting Practitioner/Parenting Leads	4 (4.0%)
Perinatal Practitioner	4 (4.0%)
Obstetrician	3 (3.0%)
Occupational Therapist	3 (3.0%)
Peer Support Worker	3 (3.0%)
Psychiatrist	3 (3.0%)
Service/Operational Manager	3 (3.0%)
Family Hubs Practitioner	1 (1.0%)
Support Worker	1 (1.0%)
Support, Time and Recovery Worker	1 (1.0%)
Other	6 (5.9%)
Missing	28

The survey initially opened in Yorkshire. Respondents with missing location data prior to the survey subsequently being opened nationwide had their location imputed as Yorkshire; missing location data after this point remains unknown. There were 101 participants with known location, of whom 79 (78.2%) were working in Yorkshire, 6 (5.9%) in the Northwest of England, 5 (5.0%) in the East of England, 4 (4.0%) in London, 4 (4.0%) in the Southwest of England, and 2 (2.0%) in the West Midlands.

The most common underserved patient groups supported by respondents included both young (< 25 years) and older (> 35 years) birthing people; ethnic or religious minorities and people with limited English language proficiency; people with social care needs, poverty or food insecurity; and birthing people with disability or neurodivergence. Approximately 75% of respondents reported that their service had made special arrangements to improve access for underserved patients, such as language translation services and tailored sessions or pathways.

**Figure 3:**
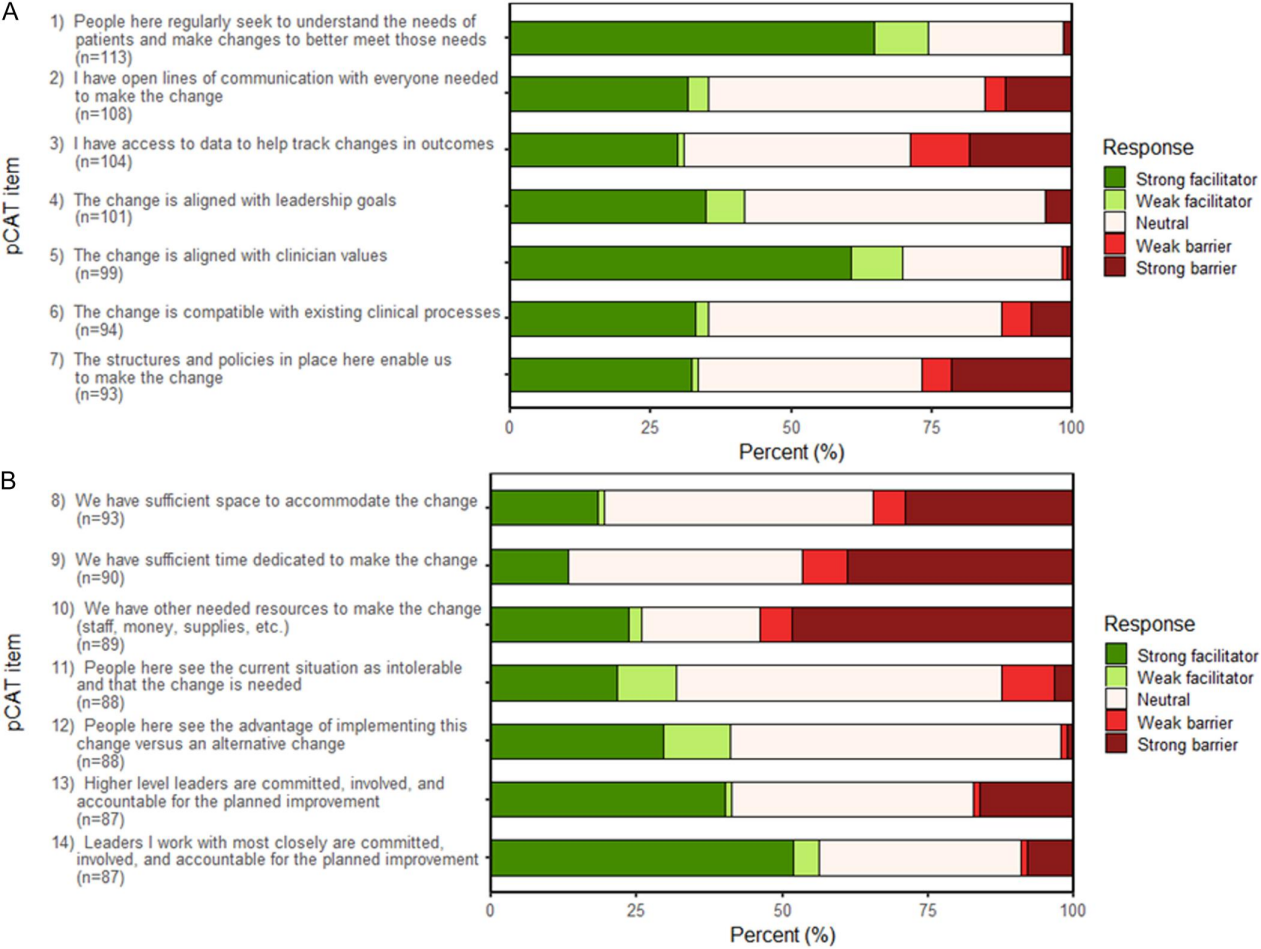
(A) pCAT responses items 1–7. (B) pCAT responses items 8–14.

The pCAT was used to identify potential facilitators and barriers to the introduction of a new perinatal mental health intervention; results of which are shown in Figure 3. Key facilitators included understanding patient needs, compatibility with clinician values, and leadership engagement. The largest barriers were identified as being available resources, including space, time, and other resources such as staff, money and supplies.

These survey findings provided a system‐level overview of implementation conditions across services, highlighting organisational facilitators (such as leadership engagement, alignment with clinician values) and constraints (particularly limited capacity and resources). These patterns informed subsequent interpretation of qualitative findings by identifying contextual factors shaping how access barriers are experienced and addressed within maternity and perinatal services.

A sub‐sample of survey respondents (*n* = 19) agreed to participate in an interview (Table 2). Survey findings were reflected in the professional interview data, where participants described similar constraints in resources, service fragmentation and workforce capacity, and provided context for women's accounts of limited access and discontinuity of care described in Work Package 2. To protect participant confidentiality within local NHS systems, professional roles are reported in grouped categories rather than specific job titles. Transcripts were coded against the candidacy framework to identify themes (Table 3).

**Table 2:** Professional groupings of interview participants (*n* = 19).

Professional grouping	*n*
Nursing and midwifery professionals (including health visitors, neonatal nurses and midwives)	5
Psychological therapy professionals	4
Medical professionals (including GPs, psychiatrists and obstetricians)	4
Service leadership and management professionals	4
Peer support and community support professionals	2

**Table 3:** Key themes identified across candidacy framework. (HCP ‐ Healthcare Professional, FG ‐ Focus Group, P‐ Participant).

Candidacy framework domain	Key themes	Supporting quotes
**Identification**	Professionals: Crisis‐point identification in vulnerable populations Window of opportunity through early outreach work Universal services (health visitors) enabling routine identification Clinical judgement essential beyond standardised tools	“Where families will want to feel like they can manage it and it's their job and their responsibility to manage this within their home or within the community. We don't often get those referrals until really late which is sad because we could have done all that early identification work” (HCP04) “I think there's quite a lot of stigma attached because the older generation, I know I've experienced it, will say don't be silly, your mother did it and she was fine or your sister doing it. You're being silly, you're being childish, there's nothing. You'll get some sort of comments as well; there's still a barrier, there's still that lack of education and awareness, I think.” (FG3)
	Experts by experience: Cultural and social barriers to help‐seeking (stigma, community norms) Lack of mental health awareness Feeling unsupported after birth trauma	
**Navigation**	Professionals: Culturally appropriate translation support critical for system access Being “in the system” as prerequisite for finding services (cycle of exclusion) Community champions breaking down access barrier Importance of language and framing (e.g. “wellbeing” vs “mental health”) Lack of joined up care Experts by experience: Not knowing what support is available/where to go for help. Navigating systems ‐ being asked about mental health can feel like a tick‐box exercise.	“I think the outreach and engagement is vital. I think… well I know that without that, we wouldn't be supporting the women that we are right now. Because we weren't supporting a number of those women prior to that outreach and engagement.” (HCP07) “Unless you know someone who's been there, how do you know about it because I was looking, clearly in the wrong places but I didn't find anything…I didn't know because I didn't know how to access that information…I think if someone had spoken to me, like my health visitor, especially in those first few weeks whether you're a first time mum or tenth time mum everything makes a difference.” (FG3)
**Permeability of services**	Professionals: Language and translation barriers Cultural matching of staff and service users Existing relationships and familiarity increasing permeability Economic barriers (travel costs, childcare) Flexible delivery formats facilitating engagement (home visits, online, phone) Experts by Experience: Feeling dismissed/not listened to Limits to when support is available Language barriers	“I've found it's really difficult to use an interpreting service ‐ the nuances of trying to explain what a therapy is and how that might be helpful is really difficult through an interpreter because I guess there's not always an alternative word for what you're trying to say is there?” (HCP01) “We found was a lot of things run out after a year, so it's like you've got a year to access X, Y or Z and it took until he was about a year old to just be able to have enough brain space to think about it so by the time we got to that year point, a lot of things had already, the opportunity had already gone…it took a long time to just think ok we can think about this now because everything else was just like firefighting all the time.” (FG4)
**Professional adjudication**	Professionals: Limitations of assessment tools/standardised measures (not fit for purpose) Complex trauma histories not fitting assessment criteria Service eligibility gaps for sub‐threshold presentations Availability of onward referral options increasing professional confidence Comprehensive handovers preserving trust and continuity Experts by experience: Parental instincts not listened to, or taken seriously Being spoken about rather than spoken to Language used, importance of meeting patients where they are, faith and cultural competency	“A lot of the screening tools are not appropriate for the groups, so you have to kind of go on more on your clinical judgement and your history‐taking, and your actual relationships” (HCP09) “A consultant came to me and he said we've done our best to stop the baby from coming and we're going to have to induce you but there's a chance, a high chance your baby may die. Now the language used by the consultant was quite damaging. And then I had another healthcare professional come in…she was a female this time and she looked at me, she understood me, she was listening and she was like listen then just trust God and everything will be okay. It's like she understood, she took the time to pause and say the right words and use the right language and it had a massive positive impact on me. She wasn't even Muslim…it's like she was culturally aware of what was important to me. “ (P017)
**Appearing at service and asserting candidacy**	Professionals: Lack of confidence or language skills to convey candidacy Fear of social services/child being taken away Experts by experience: Trust building through continuity reducing judgement fears Access to support requiring repeated advocacy Feeling discriminated against because of race Not feeling listened to	“I would certainly say there's a very cultural difference about how mental health is seen in, in certain women from certain backgrounds because they have a fear of admitting to that, either it's seen as weakness or whether they're, won't be socially accepted with them.” (HCP08) “She knew me from day one, she knew me through all my losses, she knew what my anxieties were…Like, it just made things so much easier, the continuity.” (FG2)
**Offers and resistance**	Professionals: Reduced capacity to engage due to demands of motherhood Joint appointments and integrated care models facilitating engagement Relationship‐based approach as core to engagement Direct referrals more effective than signposting Therapeutic conversations and non‐manualised creative care valued over structured programmes Experts by experience: Fear of social services/child being taken away Inappropriate support offers, that is, talking therapies Peer support and support groups are valued and beneficial	“In that perinatal period…they've got a lot on, they're just trying to cope, really. So, engaging with people and talking about things, is another thing that might feel too much for some people.” (HCP05) “I thought oh my God are social services going to come round because I'm so poorly, are they going to come and take my baby off me?” (FG3) “I went to the birth trauma group and I always say I would not be here without the birth trauma group. Absolutely would not be here, it saved my life.” (P026)
**Operating conditions and local production of candidacy**	Professionals: Involvement of partners/support networks in care Cultural competency gaps in NHS workforce Care continuity and building trusting relationships Experts by experience: Emphasis of antenatal care on physical health and not enough on emotional wellbeing of mothers. Cultural biases, institutional racism	“And we do try to have a little bit of a policy that we don't let someone fall through a gap; we would try and stay involved and get them referred to – you know, stay involved until they land somewhere else.” (HCP02) “It was very clinical, there wasn't much emphasis on me because everything was about the baby ‐ you know is the baby OK, is the baby doing well, nobody really was asking about myself” (P017) “There's a hierarchy of what is the supreme way of living or this western way of looking, living and that's the right way, that's the best way… I think there are internal faults that exist because we have different cultures and different lifestyles. I don't want to feel judged because I come from a different culture that may be different to yours” (P017)

### 4.2 Work Package 2

A total of 50 participants took part in either a focus group or interview. Participants represented a diverse range of underserved groups, including women from South Asian, Arab, Somali and Black African backgrounds, women with limited English proficiency, refugee or asylum‐seeking status, and those living in areas of high deprivation. Additional vulnerabilities included neurodivergence, caring responsibilities, physical health comorbidities and disability. Participants reported a range of mental health difficulties across the perinatal period, including birth trauma, anxiety, low mood, loneliness, relationship difficulties and low self‐esteem.

Analysis identified barriers and facilitators to PMH care across all domains of the Candidacy Framework, highlighting how access to care is shaped through ongoing interactions between women, services and wider social contexts (Table 3). While the analysis was informed by the Candidacy Framework, an inductive approach was incorporated to allow for the identification of themes extending beyond the predefined domains. This ensured that broader structural and sociocultural influences, including cultural stigma, institutional racism and community narratives, were captured and integrated into the analytical framework.

### 4.3 Triangulated Findings Across Candidacy Domains

Findings are presented thematically, integrating data from the Work Package 1 professional survey and interviews, and Work Package 2 lived experience focus groups and interviewed with underserved women and birthing people. Quantitative survey findings were used to provide contextual insight into organisational and system‐level factors shaping service provision and are integrated with qualitative findings where relevant to highlight areas of convergence and divergence across data sources.

#### 4.3.1 Identification

Across data sources, both professionals (survey and interview data) and women and birthing people (focus groups and interviews) described difficulties recognising and identifying perinatal mental health needs, particularly among underserved populations. Professionals reported that concerns were often identified at crisis points rather than earlier in the perinatal journey. Women similarly described delaying help‐seeking, often masking distress or attributing difficulties to the normal challenges of motherhood ‐ “your mother did it and she was fine or your sister doing it. You're being silly, you're being childish, there's nothing” (FG3).

Cultural stigma surrounding mental health further influenced recognition of need. In some communities, emotional distress was viewed as a private issue to be managed within families or social networks rather than through formal healthcare services. As a result, women frequently sought support only when difficulties had intensified.

Professionals emphasised the importance of early outreach and relationship‐building in facilitating identification of mental health needs. Health visitors and emotional wellbeing clinics were described as important points of contact where ongoing relationships could enable disclosure over time. However, identification practices varied across services, and some systems remained largely dependent on women self‐identifying difficulties.

#### 4.3.2 Navigation of Services

Consistent across survey findings and qualitative data, both professionals and women described significant challenges navigating perinatal mental health services. Fragmented service pathways meant that women often needed prior knowledge of available services or existing connections within the healthcare system in order to access support.

Professionals reported uncertainty about the roles and referral criteria of other services, which limited their ability to guide women effectively. Health visitors were frequently identified as key gateways to support due to their universal reach, although access still depended on women attending appointments. These findings were consistent with survey data, which indicated variable communication systems and uncertainty around service coordination, aligning with both professional and participant accounts of fragmented and difficult‐to‐navigate care pathways.

Women described similar experiences of navigating complex and poorly communicated systems ‐ “unless you know someone who's been there, how do you know about it because I was looking, clearly in the wrong places but I didn't find anything” (FG3). Several participants reported feeling that support was withdrawn abruptly following birth, leading to a sense that services “disappeared” at a time when emotional needs remained high.

#### 4.3.3 Cultural and Communication Barriers

Cultural perceptions of mental health influenced both help‐seeking behaviours and engagement with services. Professionals noted that lack of cultural representation within services could limit trust among some communities. Participants similarly described greater comfort when professionals shared cultural backgrounds or demonstrated understanding of their experiences.

Language barriers further complicated access to care. While interpreting services were recognised as valuable, participants described difficulties initiating contact with services when they lacked the language skills required to request help. Some women also reported feeling excluded from discussions about their own care due to communication barriers.

Professionals highlighted the importance of language used to describe services. In some contexts, framing support as “wellbeing” rather than “mental health” was perceived to reduce stigma and improve engagement.

### 4.4 Permeability of Services

Flexible delivery models were viewed as essential for improving access to PMH care. Home visits, online appointments and telephone consultations were seen as reducing practical barriers such as travel costs, childcare responsibilities and time constraints. Community‐based services were particularly valued for underserved populations.

Continuity of care also emerged as a key factor shaping service accessibility. Both professionals and participants emphasised that ongoing relationships with trusted professionals helped build confidence and increased willingness to disclose mental health concerns. These experiences were consistent with survey findings, which identified limited resources, including staff capacity, time and space, as key barriers to flexible service delivery, helping to contextualise professionals’ and participants’ accounts of constrained access and discontinuity of care.

Focus group participants also described fear that disclosing mental health difficulties could lead to involvement from child protection services ‐ “are they going to come and take my baby off me?” (FG3). These fears were often reinforced through community narratives and could discourage women from seeking support.

### 4.5 Professional Adjudication

Professionals described balancing clinical judgement with limitations of standardised assessment tools and service thresholds. Rigid screening criteria were sometimes perceived as insufficiently sensitive to complex social and psychological needs, particularly among women with trauma histories. Professionals highlighted that they “have to kind of go on more on your clinical judgement and your history‐taking, and your actual relationships” (HCP09).

Participants’ experiences suggested that women who were already known to services or had previous mental health histories were more likely to access support. Others described feeling dismissed or not taken seriously when attempting to communicate their struggles, particularly when communication barriers were present.

Professionals also reported that limited onward referral options constrained their decision‐making, particularly when women's needs fell between primary and secondary care thresholds.

### 4.6 Appearing at Service and Asserting Candidacy

Attending maternity or health services did not necessarily mean that women felt able to express their needs. Some participants described modifying how they presented themselves in order to be taken seriously by professionals ‐ “I find I have to do that, go in and use my best Yorkshire accent” (FG3). Others reported that assumptions about language ability or cultural background affected how their concerns were interpreted.

Cultural expectations surrounding motherhood also influenced women's willingness to disclose difficulties. In some contexts, emotional distress was framed as personal failure, leading women to conceal struggles. Fear of child protection involvement further limited disclosure.

Conversely, participants described feeling empowered when professionals validated their experiences and reassured them that seeking help did not mean they were “bad parents”.

### 4.7 Offers and Resistance

Engagement with support depended not only on availability of services but also on how support was structured. Professionals recognised that the demands of the perinatal period could make attending regular therapy appointments difficult. Some participants described structured interventions as burdensome or poorly aligned with their needs, for example for some women, weekly sessions became another “chore”.

Both professionals and women emphasised the importance of flexible, relational approaches to care. Informal support embedded within everyday settings was often preferred to highly structured therapeutic programmes.

Peer support emerged as particularly valuable. Participants described peer groups as safe spaces where they could share experiences without fear of judgement or stigma. Fear of child protection involvement also contributed to disengagement. Peer support and community relationships were often described as transformative sources of emotional support ‐ “I always say I would not be here without the birth trauma group. Absolutely would not be here, it saved my life” (P026).

### 4.8 Operating Conditions and Local Production of Candidacy

Overall, access to perinatal mental health care emerged as a dynamic process shaped by interactions between women, healthcare professionals and wider social environments. Focus group and interview participants described services as frequently focused on the baby's wellbeing, sometimes leaving maternal mental health needs insufficiently prioritised ‐ “is the baby okay, is the baby doing well, nobody really was asking about myself” (P017). Survey findings indicated that leadership engagement and alignment with clinician values were perceived facilitators to service improvement, which aligns with qualitative accounts emphasising the importance of relational and culturally responsive care.

Structural factors, including workforce diversity, cultural competence and service fragmentation, further shaped whether women felt they belonged within services. Community narratives also played a role in shaping expectations about care, with negative experiences shared within communities sometimes discouraging others from seeking support.

Overall, findings across data sources were highly convergent, with survey data identifying system‐level constraints and facilitators that were reflected in both professional and lived‐experience accounts. Together, these findings suggest that candidacy for PMH support among underserved populations is not a fixed characteristic but is produced and shaped through ongoing interactions between individuals, services and social contexts.

## 5 Discussion

### 5.1 Summary of Principal Findings

PRAMS is a mixed‐methods co‐production study using a modified Accelerated Experience‐Based Co‐Design (AEBCD) approach to explore and address inequalities in perinatal mental health (PMH) care. This paper reports findings from Work Packages 1 and 2, which captured professional and lived experience perspectives on barriers to accessing PMH care for underserved women and birthing people.

The survey component complemented qualitative insights by identifying key organisational determinants of implementation across services, particularly resource constraints, leadership engagement and perceived compatibility with existing practice and clinician values. These findings also align with and help contextualise qualitative accounts of fragmented services and capacity limitations, supporting a more integrated understanding of how structural conditions influence access to PMH care.

The findings across both Work Packages support and extend existing evidence on inequities in perinatal mental health access and service engagement, revealing that access to perinatal mental health support among underserved populations is systematically shaped by a complex interplay of structural, cultural, and relational barriers. These findings suggest that candidacy for perinatal mental health support is not a fixed individual attribute, but is actively produced through the cumulative conditions women encounter across services across every domain of the candidacy framework, and that addressing inequity in access requires structural and systemic reform, not merely improvements at the level of individual professional encounters.

### 5.2 Relationship to Other Research

Compared with existing research, PRAMS is consistent with prior evidence showing that PMH inequalities are shaped by structural, relational, and cultural barriers, including service fragmentation, workforce constraints, stigma, and lack of culturally competent care [9, 11, 16]. These studies have also highlighted persistent inequities in access for women experiencing socioeconomic disadvantage and ethnic minority women, particularly in relation to delayed help‐seeking and reduced service engagement [8]. PRAMS builds on this evidence by demonstrating how these barriers operate cumulatively across the perinatal journey, rather than at single points of contact, shaping access through dynamic processes of inclusion and exclusion across multiple service interfaces.

PRAMS advances understanding in three key ways. First, by adopting an intersectional co‐produced design across a broad range of underserved groups and mental health conditions, it moves beyond condition‐ or population‐specific analyses [23] to show how barriers interact across multiple vulnerabilities. Second, the application of the Candidacy Framework [34] extends existing work by illustrating how eligibility for care is actively produced through ongoing interactions between women and services during the perinatal period, rather than being a fixed attribute. Third, unlike much prior intervention research, PRAMS identifies system‐level redesign priorities without prespecifying a model of care, thereby shifting the focus from modifying individual services to rethinking access pathways as interconnected systems.

### 5.3 Strengths and Limitations

A key strength of PRAMS was its engagement with women and birthing people who are typically underrepresented in research and clinical services, capturing diverse perspectives across the perinatal period. Our focus group sample included those who remain entirely disconnected from existing pathways and support systems. Experiences were gathered across pregnancy, birth, and postpartum, addressing a spectrum of PMH difficulties rather than focusing on a single condition. This engagement was facilitated by the CRLW recruitment model, which fostered genuine rapport, psychological safety, and trust. As a result, participants were able to share rich, emotionally grounded narratives, ensuring that the findings reflect what matters most to underserved women and birthing people based on their lived experiences. Co‐analysis of the qualitative findings was undertaken by a team of researchers from multi‐disciplinary backgrounds and the CRLWs, which is a strength of the research.

Focus group participants from Work Package 2 reported that involvement in PRAMS enhanced confidence, connected them to supportive networks, validated their experiences, and fostered a belief that meaningful change is possible. This illustrates that co‐design can function not only as a research method but as an intervention itself, demonstrating the value of shared power, deep listening, and emotional empathy. Although the Work Package 1 survey captured a broad range of professional perspectives at a national level, the PCAT survey was not piloted with a PPIE group prior to launch. This likely contributed to incomplete responses and difficulties in interpreting items related to service “change,” potentially limiting the richness of the quantitative data. To address this, semi‐structured interviews with professionals provided in‐depth insights into service provision, drawing on a purposive sample designed for maximal variation across roles and settings. Findings from Work Package 1 are, however, based on a local sample and may reflect context‐specific pathways, policies, and operational conditions, which could limit generalisability to regions with different healthcare structures or underserved populations.

In addition, as participation was voluntary, women and professionals with particularly strong views or experiences may have been more likely to take part. Despite efforts to recruit diverse participants, some underserved groups may remain underrepresented, particularly individuals facing the greatest barriers to engagement with research and healthcare services. Although the in‐person CRLW recruitment model enabled inclusion of women who were non‐English speaking, digitally excluded, or disconnected from formal services, reliance on community‐based and relationship‐led recruitment may nevertheless have excluded individuals who were unable or unwilling to engage through these settings or networks. Consequently, findings from Work Package 2 may not therefore fully capture the experiences of those who are most socially isolated or distrustful of services and research participation. Future research could explore the value of combining trusted community recruitment with broader digital outreach strategies to maximise inclusivity.

Work Package 2 was also conducted in two local NHS contexts (Sheffield and Doncaster), and while these sites provided rich insights into system exclusion, cultural barriers, and interpreter access, the findings may not fully reflect variation in service configuration, population demographics, or the availability of culturally appropriate care across other UK regions. As such, transferability of these system‐level findings to international settings and maternity systems should be interpreted with appropriate caution.

### 5.4 Implications for Healthcare Services, Policy‐Makers and Further Research

Understanding barriers to accessing PMH care for underserved women has clear implications for service design, delivery, and policy. Findings can inform targeted changes that improve both accessibility and quality of care, enhancing professional practice and patient experiences. Services and policy‐makers can use these insights to implement coordinated, culturally sensitive, and trauma‐informed pathways for perinatal women with multiple vulnerabilities, supporting reductions in maternal mortality and improving long‐term outcomes for women, children, and families.

Findings from Work Packages 1 and 2 suggest that improving access to PMH care for underserved women requires earlier, community‐centred, and relational approaches to engagement, rather than relying on women reaching services at crisis point. The study identified how stigma, distrust of statutory services, fears around judgement or child removal, and cultural expectations that families should “manage problems themselves” can delay help‐seeking and reduce disclosure of distress. These findings suggest that future interventions should extend beyond clinical pathways alone and strengthen trusted community‐based partnerships, culturally responsive engagement, continuity of relationships, and preventative approaches aligned with wider NHS community and prevention strategies.

Work Package 3 will build on these findings through the co‐production of a system‐level intervention designed to improve trust, accessibility, and responsiveness across the perinatal care pathway for underserved women and birthing people. By targeting multiple inter‐sectionalities rather than a single ethnic group, condition, or stage of the perinatal journey, this approach aims to transform PMH pathways from fragmented and reactive systems into coordinated, proactive, and responsive care models. Improving access, experience, and timeliness of support has the potential to reduce inequalities, enhance maternal wellbeing, and promote equitable starts for underserved families.

## 6 Conclusion

PRAMS demonstrates that inequalities in access to perinatal mental health (PMH) care are produced through cumulative processes of exclusion operating across community, cultural, relational, and healthcare systems throughout the perinatal journey, rather than through isolated barriers or single service failures. By integrating the perspectives of professionals, underserved women and birthing people, this study highlights how stigma, distrust of services, fears of judgement, and cultural expectations around self‐reliance and can delay disclosure and lead many women to engage with support only at crisis point, Through its intersectional and co‐produced design, PRAMS extends current understanding of PMH inequalities by showing how candidacy for care is actively negotiated through repeated interactions with services, rather than existing as a fixed individual characteristic. Improving PMH pathways requires more than isolated interventions—it calls for system‐level, trauma‐informed redesign responsive to the diverse needs and experiences of underserved women and birthing people.

The findings also demonstrate the value of co‐production not only as a research method, but as a mechanism for surfacing hidden barriers and informing more responsive, community‐informed approaches to PMH care. These insights underscore the importance of embedding co‐production and lived experience at the heart of service design, policy, and research in perinatal mental health. Work Package 3 will build on these findings to co‐design a system‐level intervention aimed at improving trust, early engagement, and equitable access to PMH support for underserved women and birthing people.

## Author Contributions

**Elena Sheldon:** conceptualisation, investigation, funding acquisition, writing – original draft, methodology, writing – review and editing, formal analysis, project administration, data curation, supervision. **Naseeb Ezaydi:** writing – original draft, methodology, writing – review and editing, formal analysis, project administration, supervision. **Danielle Hahn:** project administration, writing – review and editing, methodology. **Kelly Hobbs:** supervision, writing – review and editing, conceptualisation, investigation, funding acquisition, resources. **Saima Ahmed:** conceptualization, methodology, project administration, supervision. **Helen Miles:** supervision, methodology, conceptualization, funding acquisition, writing – review and editing. **Julia Thompson:** writing – review and editing, investigation, conceptualization, funding acquisition, supervision, resources, project administration. **Katie Marvin‐Dowle:** supervision, writing – review and editing, conceptualization, investigation. **Kate Fryer:** conceptualization, methodology, project administration, supervision, writing – review and editing. **Laura Sutton:** formal analysis, writing – original draft, conceptualization, funding acquisition, data curation. **Victoria Silverwood:** conceptualization, investigation, funding acquisition, project administration, supervision, writing – review and editing. **Caroline Mitchell:** writing – review and editing, project administration, supervision, methodology, funding acquisition, conceptualization, investigation. **Daniel Hind:** conceptualization, investigation, funding acquisition, writing – review and editing, methodology, project administration, supervision. **Kelly Mackenzie:** conceptualization, investigation, funding acquisition, project administration, formal analysis, supervision, resources, writing – review and editing, methodology.

## Ethics Statement

Ethical approval was granted by the Health Research Authority and Health and Care Research Wales Ethics Committee (27 January 2025/24/PR/1495).

## Conflicts of Interest

The authors declare no conflicts of interest.

## Supporting information


Supporting File 1

Supporting File 2

Supporting File 3

## Data Availability

The data sets used and/or analysed during the current study are available from the corresponding author upon reasonable request.
