# Supplementary material for: Co‐Producing Equitable Perinatal Mental Health Care: Facilitators and Barriers to Access Among Underserved Women in the PRAMS Study
Source: Health Expect. 2026 Jul 12;29(4):e70766. doi: 10.1111/hex.70766 (PMC13356889; doi:10.1111/hex.70766)

Tab 1

**Risk Protocol: Perinatal Redesign for Accessing Mental Health Services (PRAMS)**

**Policy Statement**

GPs are responsible for the ongoing clinical care of study participants. Therefore, researchers, including qualitative researchers, and Community Research Link Workers (CRLWs) have a duty of care to ensure that the GP is aware of suicide ideation expressed by study participants.

The PRAMS researcher and/or CRLW will initiate the suicide ideation protocol each time a study participant expresses thoughts of suicide or self-harm. This may be as a result of a study participant disclosing information during an interview, focus group or co-design workshop that leads the researcher to believe that there is a suicide risk. This may also be a result of engagement with eligible women or birthing people in community settings prior to recruitment into the study. In this instance, the researcher or CRLW, with the study participant’s permission, should inform the study participant’s GP and notify the Project Management Group (PMG).

If the study participant refuses permission for the researcher to inform the GP then the researcher should immediately consult the nominated clinician in the PMG, who will then examine the study participant’s circumstances and, if necessary, will assess the study participant. If it is concluded that there is a significant risk, the study participant’s GP will be notified **with or without** the study participant’s consent. However, the nominated clinician would contact the GP without first assessing the study participant her/himself if the situation was urgent, again with or without the study participant’s consent. In these cases, the decision should be explained to the study participant as soon as possible.

The CRLW or researcher may also wish to use Supervision and Reflective Practice space offered by the Clinical Psychologist and Assistant Psychologist as part of the project delivery. This space can be used to bring cases that may have caused concern for the researcher or CRLW but they are unsure of how to proceed in terms of signposting to appropriate support for the identified mental health need.

**Definition of suicide ideation**

In this study, suicide ideation is identified by:

- Study participant discloses information during an interview, focus group or co-design workshop to a member of the research team, indicating that they have attempted suicide or that they have been thinking of ways to die by suicide.

In this instance the suicide ideation pro forma (end of document) should be completed to convey these thoughts to the study participant’s GP and to record the incident as detailed in action required. However, if the researcher or CRLW believes for any other reason that the study participant is at risk of self-harm, they should contact the clinical members of the PMG to discuss.

A schematic of the suicide ideation pro forma is shown in figure 1.

Before each contact with a study participant (either telephone or face-to-face), the researchers or CRLW should review all previous data on suicide ideation and ensure that contact details for the nominated clinician in the PMG are current.

When the PRAMS researcher or CRLW becomes aware that a study participant has thoughts of suicide, they should first of all ascertain whether or not the study participant has talked to their GP about them. The researcher or CRLW should reinforce the importance of the study participant maintaining a dialogue with their GP and ask for permission to pass the information to the study participant’s GP. Suggested scripts are shown below. If the study participant agrees to this communication, the researcher should inform the study clinician who will telephone the study participant’s GP within 48 hours** to pass on the information obtained.

If the study participant’s GP is not available then the study clinician should ask to speak to the duty doctor. The study clinician should make it clear to the GP that no risk assessment has been performed and that clinical responsibility for the study participants remains with the GP. An email should be sent to the GP confirming this notification.

If the study participant does not agree to their GP being informed, the study clinician should discuss this with the PMG and where risk severity is significant or risk is urgent, the study participant’s GP should be contacted **with or without** the study participant’s consent.

Please note: If the researcher conducting the interview is [anonymised] they will act in her capacity as a study clinician and will directly contact the patient’s GP.

**If the researcher believes the study participant is in immediate danger, the researcher must immediately contact the study clinician, who will take appropriate action. For urgent crisis support, the researcher or CRLW should refer to NHS 111 option 2.

**Face-to-face/telephone interview, focus group (WP2) or workshop (WP3) with participant**

- If the study participant discloses information regarding suicide intent the researcher should discuss the importance of informing their GP using the suggested scripts below
- If the study participant agrees to GP being informed, the researcher should action the suicide ideation pro forma as detailed above
- If the study participant does not agree to GP being informed, or if GP cannot be contacted within 48 hours, the researcher should discuss with the nominated clinician in the PMG and if urgent, refer to NHS 111 option 2

***Suggested Scripts:***

1. ***Disclosure during 1:1 interview, focus group or workshop***

*I am concerned about some of the things you have told me. Have you spoken to your doctor about them? It is important that your doctor knows about the way you feel, as they will be able to make sure that you have the necessary support in place. Are you happy for me to mention to your doctor the things you have told me?*

1. ***If study participant is hesitant or refuses***

*Many people find it hard to bring these things up during a consultation, but your GP can offer you help with these feelings. If he/she knows how you are feeling, he/she will be able to talk to you about it and together you can decide on the best way to treat you.*

1. ***If study participant continues to refuse***

*That's fine, but as I am not a medical doctor, I do have to let my colleague know about the way you are feeling. They may phone you in the next day or so to have a talk to you about the way you are feeling. Risk Protocol v1.0 15.11.2024 Figure 1 – Suicide ideation pro forma The following action must be taken and recorded by a member of the research team whenever a study participant discloses suicide ideation to a researcher or CRLW.*

**Figure 1 - Suicide ideation proforma**

The following action must be taken and recorded by a member of the research team or CRLW whenever a study participant discloses suicide ideation.

**
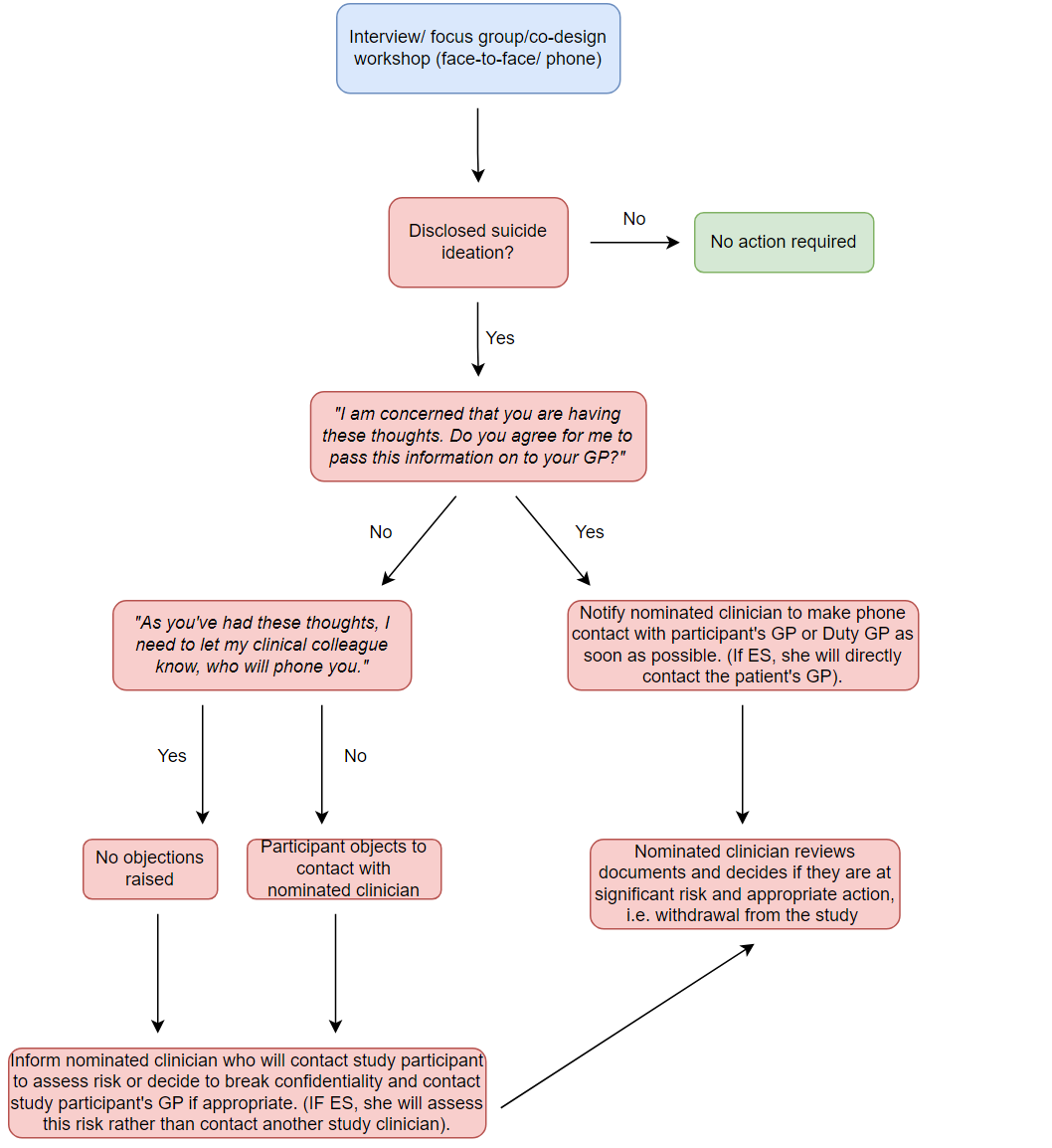
**

**Appendix: GP Letter**

GP name

Address line 1

Address line 2

Address line 3

Date

Research Reference Number

**FAO GP of participant in PRAMS research study**

Dear Doctor,

**The PRAMS study: Perinatal Redesign for Accessing Mental Health Services**

**Notification of suicide ideation**

**Patient’s name: ________________ Date of Birth: _______________**

The above patient is taking part in our study using Experience-Based Co-Design to improve access to perinatal mental health care for women in underserved groups.

I am writing to notify you that the above patient reported thoughts of suicide ideation at **[state time period]** today.

The patient disclosed the following information during **[an/a interview/focus group/workshop]** with a researcher **[in person/over the phone]:**

**[Researcher to add detail]**

We would like to remind you that no risk assessment has been carried out as part of the study, and ongoing clinical care remains with you. It is part of our study protocol to inform you of such risks, so that you can take account of them in your care plan.

Yours Sincerely,

**[Nominated clinician]**


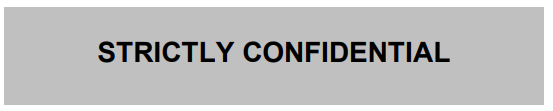

Supplement: Supplementary file 2 — Supporting File 2 [file HEX-29-e70766-s001.docx]
