# Supplementary material for: Co‐Producing Equitable Perinatal Mental Health Care: Facilitators and Barriers to Access Among Underserved Women in the PRAMS Study
Source: Health Expect. 2026 Jul 12;29(4):e70766. doi: 10.1111/hex.70766 (PMC13356889; doi:10.1111/hex.70766)
Supplement: Supplementary file 3 — Supporting File 3 [file HEX-29-e70766-s002.docx]

**Suggested template safeguarding plan**

This suggested form can be used to help you consider the safeguarding issues relevant to you project and to document how you will address them. This may be of particular use for projects which do not require ethical review as they do not involve human participants, personal data or human tissue. Keep your safeguarding plan under regular review and update it as required.

| **Project title:** **PRAMS (Perinatal Redesign for Accessing Mental health Services)** |
| --- |
| **sPrincipal Investigator: [anonymised]** |
| **Which groups either involved in, or potentially affected by, the project need to be considered for safeguarding purposes?** *Internal research team (staff/students)? External research team/members of partner organisations? Research participants? Others who may be affected by the research activities (e.g. members of research participants’ families/households; members of broader communities)?*  **Internal research team**  **Research partners: [anonymised].**  **Research Participants, including healthcare professionals (work packages 1 and 3) and women from underserved groups with lived experience of perinatal mental health problems (work packages 2 and 3).** |
| **For each relevant group identified (delete sections if not relevant):** |
| 1. **Internal research team members (University of [anonymised] staff/students):** |
| **What risks of harm are relevant for this group?** *(Risk of harm encompasses all forms of injury or abuse including bullying, exploitation, psychological abuse, physical violence, and any sexual exploitation, abuse or harassment).*  Internal research team members are exposed to the general risks of abuse, exploitation,  and misconduct associated with undertaking research. Beyond this, the specific risks  associated with this project are the following:  • Possible distress hearing about patient experiences  • Completing face to face focus groups and events with participants in a community setting |
| **How will these risks be mitigated as far as possible?** *(This might include: informing individuals of their responsibilities under the Preventing Harm in Research & Innovation (Safeguarding) policy and setting clear expectations regarding conduct across the research team; undertaking a departmental risk assessment; applying for ethics approval where relevant; implementing a lone worker policy where relevant; provision of relevant training; ensuring appropriate insurance is in place, undertaking DBS checks or equivalent where relevant; following the Security Sensitive Research Policy and Process where relevant, etc.)*  Responsibilities under the Preventing Harm in Research & Innovation Safeguarding policy.  Setting clear expectations regarding conduct across the research team.  Study risk assessment log which is regularly reviewed.  Lone worker policy, completing the ‘lone worker checklist’ for conducting any focus groups and co-design events.  Provision of relevant online training by The University of [anonymised].  Signposting to mental health support as appropriate by healthcare professionals in the team.  Introduce wellbeing check-ins and check-outs as part of internal team meetings, external meetings (PPI meetings, PSC meetings), focus groups, 1:1 interviews, and co-design events to help consistently foster an open culture and a level of psychological safety amongst staff and people with lived experience more broadly. |
| **How will individuals be informed of the mechanisms available to them for reporting an incident or concern?** *(See policy sections 5.1-5.3).*  Safeguarding Policy - provides information and contact points for staff on ensuring the  welfare of children and vulnerable adults in the University community.  Dignity at Work toolkit - information and guidance for staff on investigating and responding  to allegations of bullying, harassment, discrimination and victimisation.  Report + Support - available to any member of staff, student, or visitor for reporting of any  form of discrimination such as harassment, abuse, bullying, or sexual violence that may be  based on race, gender, sexuality, or other characteristics.  Public Interest Disclosure (Whistleblowing) policy - available to all employees and workers  for raising matters of concern that are in the public interest (often referred to as  whistleblowing), such as: malpractice, impropriety or wrongdoing. Such disclosures may  be about the alleged wrongful conduct of the University, or about the conduct of a fellow  employee, service user, or any third party. |
| 1. **External research team/members of partner organisations:** |
| *The below should be discussed openly and sensitively with partner organisations, and an agreed approach documented in writing.* *Where relevant, plans should be also be developed in consultation with other stakeholders and communities.* |
| **What risks of harm are relevant for this group?** *(Risk of harm encompasses all forms of injury or abuse including bullying, exploitation, psychological abuse, physical violence, and any sexual exploitation, abuse or harassment).*  External research team/members of partner organisations, Community Research Link Workers (CRLWs), and the Patient and Public Involvement (PPI) panel members, are exposed to the general risks of abuse, exploitation, and misconduct associated with undertaking research. Beyond this, the specific risks associated with this project are the following:  • Possible distress hearing about patient experiences  • Possible distress disclosing personal experiences, if relevant, by the PPI panel or CRLWs with lived experience  • Possible distress when supporting women or birthing people with an unmet mental health need and signposting to appropriate support |
| **How will these risks be mitigated as far as possible?** *(This might include: setting clear expectations regarding conduct across the research team and responsibilities to report incidents/concerns; undertaking a departmental risk assessment, applying for ethics approval where relevant; implementing a lone worker policy where relevant; provision of relevant training; ensuring appropriate insurance is in place, undertaking DBS checks or equivalent where relevant etc.)*  Setting clear expectations regarding conduct across the research team and  responsibilities to report incidents/concerns.  Applying for ethics approval for the research study.  Study risk assessment log which is regularly reviewed.  Signposting to mental health support as appropriate by healthcare professionals in the team.  Clinical members of the Project Management Group will offer a regular supervision and reflective practice space to CRLWs, PPI members and/or LIGHT peer support workers to discuss any cases that raise concerns about risk or safeguarding.  A risk protocol will be in place outlining the process to follow if suicidal dietitian, self harm or a mental health related risk is identified in a community or research setting.  Complex cases will be assessed on a case by case basis internally by members of the PMG with clinical expertise to support appropriate signposting.  Two mental health professionals (KH and ES) will provide psychologically informed training to the CRLWs to support them in screening and recruitment, specifically in identifying risk factors, managing safeguarding concerns and disclosures, signposting or referral support; engaging with participants, controlling group dynamics, listening skills, and safeguarding. |
| **Who will be the Designated Safeguarding Contact(s) (DSC) in the research team?** *(For research undertaken outside the UK, ideally DSCs should be based in the relevant country/ies).*  CTRU Lead for the study,  [anonymised] |
| **What other route(s) (e.g. an alternate DSC outside the research team) will be available to the external members of the research team if they need to report an incident or concern?** *(At least one route should be clearly independent of the research team, e.g. Head(s) of relevant academic department(s), senior member(s) of partner organisation(s); University of [anonymised] Research Ethics & Integrity Manager).*  The University’s Research Ethics and Integrity Manager  [anonymised] |
| **How will all relevant individuals be informed of the mechanisms available to them for reporting an incident or concern?**  Circulating the Safeguarding Plan to the Project Management Group and PPI panel for their information.  Setting clear expectations regarding conduct across the research team and  responsibilities to report incidents/concerns in team meetings and in the Terms of  Reference document. |
| **How will reported incidents/concerns be handled and escalated?** (*This should take into consideration the regulatory, statutory or legislative frameworks applicable to the partner organisation(s) and where the research is being carried out, as well as the requirements of the University’s policy on Preventing Harm in Research & Innovation (Safeguarding).*  Incidents can be reported to the Designated Safeguarding Contact (DSC) or the University’s Research Ethics and Integrity Manager by email or telephone.  The procedure for dealing with reported concerns or incidents is as follows:   - Where possible, the DSC should address any immediate safety concerns and/or offer appropriate immediate support to the individual(s) raising the concern, in accordance with the context-specific action plan for handling such concerns agreed as part of the planning process, whilst taking care to set clear expectations regarding the level and type of support that they are able to offer. - The DSC should try to obtain as much information as possible regarding the reported concern, whilst also acting sensitively and providing reassurance regarding the process that will be undertaken for handling the report. Where possible, information regarding the action that the victim would like to see taken in response to the incident (and also any actions that they would prefer not to be taken) should be sought, so that this can be considered as a central concern in addressing the matter. - The DSC should notify Naseeb Ezaydi as the Project Manager for the study (unless they are the subject of the concern); the Head of the relevant department; and the NIHR as the funding organisation. These parties should be notified as soon as reasonably practicable so that steps can be taken to prevent or limit any immediate or or-going risks of harm (for example pausing the research & innovation activity), and to enable the matter to be investigated. - At this stage, the Research Ethics & Integrity Manager in Research Services should also be notified so that advice can be sought regarding the appropriate next steps (e.g. referral to another appropriate University policy or procedure; commencement of a fact-finding process, convening of a specific safeguarding investigation panel to consider the matter; decision not to proceed with investigating the matter in response to victim’s wishes or insufficient information being provided). Advice may be sought from one of more the following, and/or an investigation panel may include members of the following, depending on the nature of the concern:   - The University’s Safeguarding Panel   - Human Resources   - University Research Ethics Committee   - Vice-President for Research and/or Faculty Directors of Research & Innovation   - Members of partner organisations involved in the research. - The victim, and other relevant parties should be kept informed regarding progress and key decisions in dealing with the matter. - If at any point, it is identified that the reported concern comes under the remit of another University policy or procedure, it should be referred to be handled under that policy or procedure. This may include the following:   - Safeguarding Policy;   - Disciplinary Procedures for staff or students;   - Procedure for Investigating and Responding to Allegations of Research Misconduct;   - Procedure for investigating concerns in relation to compliance with the University’s Ethics Policy Governing Research Involving Human Participants, Personal Data and Human Tissue. - If the University has reason to believe that a crime has been committed or that there is imminent risk of harm occurring, a report should be made to the appropriate authority, except in circumstances where there is a reasonable expectation that to do so would cause further harm to the victim/survivor. - In some cases, where the matter has been reported by a third party/witness, the victim may not wish to provide testimony, and/or they may not wish the investigation to proceed. Consideration should be given to this, particularly where there is a risk of further harm as a result of the investigation proceeding. However, in making a decision regarding whether to proceed with an investigation, the potential for future abuse and harm must be considered, along with national and international law. - In addressing a reported concern, attention should be paid to how the situation arose, and actions which should be taken at an individual, team, departmental or organisational level to try and ensure that a similar concern does not arise again in future. - Where required, in accordance with funding body terms and conditions, Research Services will liaise with the department concerned to ensure that concerns or incidents are reported to the funder at the appropriate timepoints. |
| 1. **Research participants: (NB. If your research involves research participants you will need to obtain** [**ethics approval**](https://www.sheffield.ac.uk/rs/ethicsandintegrity/ethicspolicy/approval-procedure/routes)**. If applying for University of [anonymised] ethics approval you will be able to consider and document your safeguarding plan as part of your ethics application so you will not need to maintain a separate plan)** |
| **What risks of harm are relevant for this group?** *(Risk of harm encompasses all forms of injury or abuse including bullying, exploitation, psychological abuse, physical violence, and any sexual exploitation, abuse or harassment).*  Research participants are exposed to the general risks of abuse, exploitation, and misconduct associated with undertaking research. Beyond this, the specific risks associated with this project are the following:  • Possible distress disclosing information about their professional or patient experience and relationship with health services |
| **How will these risks be mitigated as far as possible?** *(This might include: setting clear expectations regarding conduct across the research team; consultation with key stakeholders/representatives of communities to be involved in the research; undertaking a departmental risk assessment, applying for ethics approval (considering key issues such as  power dynamics; informed consent arrangements; maintaining participant confidentiality, feedback of research results); provision of relevant training for researchers; undertaking DBS checks or equivalent where relevant etc.).*  Consultation with the study PPI Panel which will include 2-3 representatives with lived experience; specifically the review of patient-facing materials such as information sheets.  Applying for NHS ethics approval which considers informed consent arrangements; maintaining  participant confidentiality, feedback of research results.  Provision of relevant training for researchers, including Community Research Link Workers who have been embedded within underserved community to support recruitment and engagement. This training will be developed by clinically trained members of the team and will ensure that researchers have an understanding of risk factors, safeguarding processes, how to make a referral and how to signpost to support services. This training will be provided to all members of the team who will be working with research participants in work packages 2 and 3.  Provision of information for contacting support services such as Talking Therapies, Samaritans, Peer Support and crisis services, in the information sheet.  Focus groups and co-design events will be developed and/or co-facilitated by a qualified Clinical Psychologist, an Assistant Psychologist, and a Peer Support worker, all of whom have experience in supporting women with perinatal mental health problems. Whilst the focus groups and co-design events are not therapy and we do not expect individuals to share lots of personal information about historical or ongoing trauma that is not relevant to the aims of the focus group.  The PMG will liaise with colleagues across wider regional services, such as Doncaster and Rotherham perinatal mental health services and Talking Therapies, to ensure awareness of the project and support with referrals and signposting should a need be identified through conducting the research. |
| **Who will be the Designated Safeguarding Contact(s) (DSC) in the research team if not already documented in previous section?** |
| **What other route(s)/mechanism(s) (e.g. an alternate DSC outside the research team) will be available to the research participants if they need to report an incident or concern if not already documented in previous section?** *(At least one route should be clearly independent of the research team, e.g. a trusted member of the researched community, Head(s) of relevant academic department(s), senior member(s) of partner organisation(s), University of [anonymised] Research Ethics & Integrity Manager). Contacts and mechanisms for reporting should be appropriate for the participants and ideally agreed through consultation with representatives of researched communities (mechanisms for reporting may include email, phone, social media, comments boxes (potential for anonymous reporting should be considered). Careful consideration should be given to the potential barriers to reporting of concerns and how these can be addressed. Potential barriers may include real or perceived power imbalances, language barriers, and fear of retribution/negative consequences. For example, community members may feel more comfortable with reporting a concern to a community leader with whom they are already familiar (who will then escalate the concern in accordance with a defined procedure), or via an anonymous reporting mechanism (either virtual or physical) rather than having to directly contact a member of the research team.*  The University’s Research Ethics and Integrity Manager [anonymised] |
| **How will all participants be informed of the mechanisms available to them for reporting an incident or concern?** *(E.g. via participant information sheets, posters, web pages, oral delivery of information at community talks).*  Information and contact details provided on participant information sheets and the  quarterly participant newsletter |
| **How will reported incidents/concerns be handled and escalated, if not already documented in previous section?** (*This should take into consideration the regulatory, statutory or legislative frameworks applicable where the research is being carried out, as well as the requirements of the University’s policy on Preventing Harm in Research & Innovation (Safeguarding)).* |
| 1. **Others who may be affected by the research activities (e.g. members of research participants’ families/households; members of broader communities; other external stakeholders e.g. organisations who are not formal project partners, attendees at a public engagement event)** |
| **What risks of harm are relevant for this group?** *(Risk of harm encompass all forms of injury or abuse including bullying, exploitation, psychological abuse, physical violence, and any sexual exploitation, abuse or harassment).*  **N/A** |
| **How will these risks be mitigated as far as possible?** *(This might include: setting clear expectations regarding conduct across the research team; undertaking a departmental risk assessment, consultation with key stakeholders/representatives of communities to be involved in the research regarding how best to communicate mechanisms for reporting concerns; applying for ethics approval where relevant, provision of relevant training for researchers; undertaking DBS checks or equivalent where relevant etc.), making appropriate provision at events for those who may have medical conditions/disabilities)*  N/A |
| **Who will be the Designated Safeguarding Contact(s) (DSC) in the research team if not already documented in previous sections?**  **N/A** |
| **What other route(s)/mechanism(s) (e.g. an alternate DSC outside the research team) will be available to individuals if they need to report an incident or concern, if not already documented in previous section(s)?** *(At least one route should be clearly independent of the research team, e.g. a trusted member of the researched community, Head(s) of relevant academic department(s), senior member(s) of partner organisation(s), University of [anonymised] Research Ethics & Integrity Manager). Contacts and mechanisms for reporting should be appropriate for the research community and ideally agreed through consultation with relevant representatives/stakeholders (mechanisms for reporting may include email, phone, social media, comments boxes (potential for anonymous reporting should be considered). Careful consideration should be given to the potential barriers to reporting of concerns and how these can be addressed. Potential barriers may include real or perceived power imbalances, language barriers, and fear of retribution/negative consequences. For example, community members may feel more comfortable with reporting a concern to a community leader with whom they are already familiar (who will then escalate the concern in accordance with a defined procedure), or via an anonymous reporting mechanism (either virtual or physical) rather than having to directly contact a member of the research team.*  N/A |
| **How will individuals be informed of the mechanisms available to them for reporting an incident or concern, if not already documented in previous section(s)?** *(E.g. posters, web pages, oral delivery of information at community talks).*  **N/A** |
| **How will reported incidents/concerns be handled and escalated, if not already documented in previous section?** (*This should take into consideration the regulatory, statutory or legislative frameworks applicable where the research is being carried out, as well as the requirements of the University’s policy on Preventing Harm in Research & Innovation (Safeguarding)).*  *N/A* |
